# Antimicrobial prophylaxis for vesicoureteral reflux: which subgroups of children benefit the most?

**DOI:** 10.21203/rs.3.rs-3286108/v1

**Published:** 2023-08-30

**Authors:** Beibo Zhao, Anastasia Ivanova, Nader Shaikh

**Affiliations:** The University of North Carolina at Chapel Hill Gillings School of Global Public Health; The University of North Carolina at Chapel Hill Gillings School of Global Public Health; University of Pittsburgh

**Keywords:** Risk, efficacy, prophylaxis, urinary tract infection, Vesicoureteral Reflux, bowel-bladder dysfunction

## Abstract

**Background:**

While the Randomized Intervention for children with Vesicoureteral Reflux (RIVUR) trial found that long-term antimicrobial prophylaxis reduced the risk of urinary tract infection (UTI) recurrences by 50%, 10 children had to be treated with long-term antimicrobial prophylaxis for one to benefit (i.e., observed number needed to treat (NNT) of 10). Accordingly, we re-analyzed RIVUR data to systematically identify subgroups of children with vesicoureteral reflux (VUR) with a smaller NNT.

**Methods:**

Using patient-level data from the RIVUR trial, we applied penalized regression methods including the baseline age, VUR, and bowel-bladder dysfunction (BBD) as covariates to identify subgroups that consider the trade-off between absolute risk difference and size.

**Results:**

We identified three relevant subgroups of children that appear to benefit from long-term antimicrobial prophylaxis, all with NNTs smaller than the NNT of 10. Children with grade IV VUR and BBD, 1% of the RIVUR sample, had a NNT of 2; children with BBD, 12% of the RIVUR sample, had a NNT of 4; children with BBD (and any grade VUR) or with grade IV VUR (regardless of BBD status), which was the combination of the first two subgroups and included 19% of children in the RIVUR sample, had a NNT of 4.

**Conclusions:**

Use of long-term antimicrobial prophylaxis appears to be particularly relevant for children with BBD (and any grade of VUR) or those with grade IV VUR (regardless of BBD status) who were at high risk of UTI recurrences.

## BACKGROUND

The Randomized Intervention for children with Vesicoureteral Reflux (RIVUR) trial was a multisite, randomized, placebo-controlled trial aimed at evaluating the efficacy of antimicrobial prophylaxis in preventing recurrences of urinary tract infection (UTI) in children with vesicoureteral reflux (VUR) diagnosed after an index UTI [1]. A total of 607 children 2 to 71 months of age were randomized in the ratio of 1:1 to receive either antimicrobial prophylaxis or placebo daily and were followed for 2 years. The study found that long-term antimicrobial prophylaxis reduced the risk of UTI recurrences by 50% (23.6% vs. 12.9%, corresponding to a observed number of needed to treat (NNT) of 10) [2]. Because use of long-term antimicrobial prophylaxis may lead to the development of antibiotic resistance and alterations of microbiome, and because the number needed to treat observed in the RIVUR trial was relatively large, routine use of long-term antimicrobial prophylaxis for all grades of VUR remains controversial.

Accordingly, there is an interest in identifying higher-risk subgroups of children that would benefit the most from long-term antimicrobial prophylaxis. Two previous studies have used patient-level data from the RIVUR trial to examine subgroups of children for whom long-term use of antimicrobial prophylaxis could be advocated. Wang et al. (2018) used the data from the RIVUR study to test the performance of a model developed in a previous relatively small study in a referral population [3]. Shaikh et al. (2020) used a cost-utility model to evaluate the trade-off between benefits and risks of prophylaxis based on VUR grade and concluded that treating children with grade IV VUR is cost effective [4]. While helpful in clarifying the clinical and financial trade-offs in children with VUR, the latter study did not explicitly take into account covariates other than grade of VUR.

In this manuscript, we sought to further explore data from the RIVUR trial to identify subgroups of children with VUR who appear to benefit the most from long-term antimicrobial prophylaxis. We used penalized logistic regression methods to systematically identify subgroups that consider both the treatment effect in the subgroup and the size of the subgroup.

## METHODS

### Analytic Sample

The study design and data collection of the RIVUR trial has been described previously [1]. In the RIVUR trial, 39 of 302 children (event rate, 0.13) had a recurrent UTI in the antibiotic prophylaxis treatment arm (T) compared to 72 of 305 children (event rate, 0.24) in placebo control arm (C). Five children with missing VUR grade were excluded from the analysis, leaving an analytic sample of 602 children. A total of 38 children in the treatment arm and 42 in control arm were lost to follow-up before their final visit (2 years after enrollment) and did not have a UTI recurrence while in the trial. Because 486 of 607 children were followed for at least one year without a recurrence, and because most observed recurrences occurred within the first year (the interval between enrollment and a 10% incidence of recurrence was 336 days and 106 days in the treatment arm and the control arm, respectively) [2], we analyzed these participants as without an event following the example of the original analysis undertaken in the trial.

### Covariates

Baseline demographic and clinical characteristics of the RIVUR trial participants have been reported previously [1]. For this subgroup analysis, we included all three covariates assessed at enrollment that had a p-value for interaction with treatment of less than 0.1 [2]: index UTI type (febrile or symptomatic), grade of VUR (I-IV), and bladder and bowel dysfunction (BBD). Because BBD was not assessed in children 2–23 months of age, we created a composite covariate that combined age and BBD. This composite covariate had four levels: (1) age 2–23 months and not toilet trained; (2) age 24–71 months and not toilet trained; (3) age 24–71 months and toilet trained with BBD absent; and (4) age 24–71 months and toilet trained with BBD present. Risk factors used in creating our model have been identified in previous studies [5–8].

### Statistical methods

First, we generate a pool of candidate subgroups through thresholding linear predictors of fitted penalized logistic regression models with treatment, covariates, covariate-by-covariate interactions, and treatment-by-covariate interactions. More information on model fitting, variable selection methods, and generation of candidate subgroups is available in the **Supplementary Materials**. Of note, we require all candidate subgroups containing children with low grade VUR to also contain children with higher grades VUR, because, from a clinical perspective, if children with low grade VUR benefit from prophylaxis, so would those with higher grade VUR. Similarly, we require all candidate subgroups including toilet trained children without BBD to also include toilet trained children with BBD.

From the generated pool of candidate subgroups, we identify subgroups that balance the trade-off between the magnitude of the treatment effect and the size of the subgroup so that relatively small subgroups that exhibited beneficial treatment effect, measured by large absolute risk difference of recurrent UTI between the placebo control arm and the prophylaxis treatment arm (hereinafter referred to as risk difference), could be identified. Because antibiotic use can have negative consequences, we prioritize identification of small subgroups with a number needed to treat lower than the overall sample. Following methods developed by Zhang et al. [9], we identify subgroups through maximizing the risk difference in the subgroup multiplied by the prevalence of the subgroup raised to a power term. Varying the power term from 0 to 1.5 with an interval of 0.001 allowed us to identify a list of such subgroups, from the smallest subgroup with the largest risk difference to the largest subgroup with the smallest risk difference. More information is available in the **Supplementary Materials**.

For each subgroup, we calculated the observed NNT. However, because this can result in overly optimistic estimates of the true NNT in these subgroups [10 11], we also present results obtained by using a permutation-based cross-validated (CV) procedure (with two-fold CV and 100 permutations) which attempts to correct this potential bias [9], Details on the procedure are provided in the **Supplementary Materials**. We performed all statistical analyses using R [12].

## RESULTS

**Figure 1.**
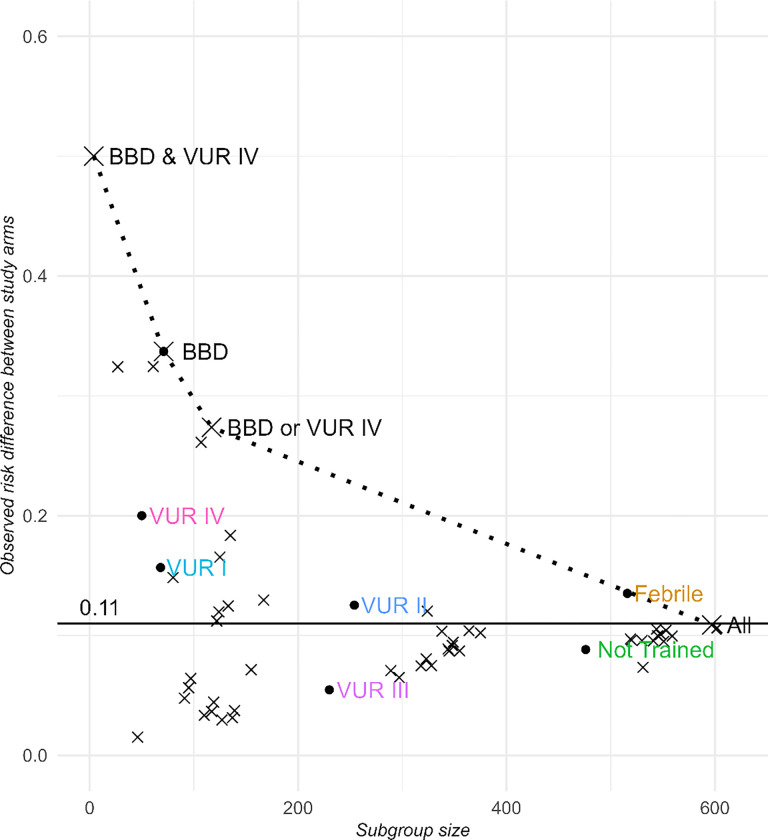
Candidate subgroups identified via penalized regression with a positive observed risk difference plotted with X symbols according to subgroup size. The best subgroups are labelled with enlarged symbols and connected by a dotted line. The horizontal line at 0.11 is the observed risk difference in all participants. Other clinically relevant subgroups are also labeled in colored font. Detailed methods used to identify candidate subgroups are available in Supplementary Materials.

Characteristics of subgroups identified are presented in Table 1. The subgroup of children with grade IV VUR and BBD (observed NNT = 2), representing less than 1% of the children in the RIVUR sample, appear to have benefited the most from long-term antimicrobial prophylaxis. This is followed by a subgroup of children with BBD, 12% of the RIVUR sample with an observed NNT = 4. The third subgroup, which is a combination of the first two subgroups, includes children with BBD (and any grade of VUR) or those with grade IV VUR (regardless of BBD status). This subgroup comprises 19% of the RIVUR sample with an observed NNT of 4. In addition to the observed NNT, the adjusted NNT is also reported in Table 1. Notably, the subgroup of children with grade III VUR who did not have BBD, which comprised 45% of the RIVUR sample, had an observed NNT of 24, which is why this subgroup was not selected. Figure 1 plots the identified subgroups and candidate subgroups who had worse trade-offs and were therefore not selected.

## DISCUSSION

We found that children with BBD (and any grade of VUR) or grade IV VUR (regardless of BBD status) appear to benefit the most from long-term antimicrobial prophylaxis. This subgroup appears to have been driving the differences in the risk of recurrent UTI in the RIVUR trial. The observed NNT in this subgroup was 4 [13].

Our results are generally consistent with previous studies but provide a clearer picture of the subgroups that may benefit from treatment. A previous cost-effectiveness analysis considering only grade of VUR as the only covariate, found that long-term antimicrobial prophylaxis appeared to be most cost effective in children with grade IV VUR [4]. While Wang et al. [14] suggested that females and uncircumcised males with both grade I-III VUR and BBD are also at high risk, these risk factors were selected was based on data from a relatively small study performed in a referral population, not patient-level data from the RIVUR study [3]. Kent and Hayward have noted that treatment effect is usually higher in subgroups with higher risk [15]. This was true in our data; the best subgroups we identified also had the highest rate of UTIs.

What do our results mean for the practitioner? Shaikh et al. [4] suggested that treatment of children with grades IV was clearly cost-effective. While our results also support treatment of children with grade IV VUR, we found that, in addition to children with grade IV VUR, those with lower grades of VUR who have BBD also appear to derive substantial benefit from antimicrobial prophylaxis. Both this study and the study by Shaikh et al. suggest that treatment of children with grade III VUR who do not have BBD might not be particularly effective.

There are several limitations in our investigation. First, the RIVUR trial was not powered to detect treatment effect heterogeneity [2]. Second, there were very few children with grade IV VUR (8.3% of the RIVUR sample). Third, the RIVUR study excluded children with grade V VUR. Fourth, our results are only directly applicable to children similar to those enrolled in the RIVUR trial [16]. Because changes in imaging following the 2011 American Academy of Pediatrics guideline on the management of young children with UTI [17] may have changed the distribution of children with various grades of VUR, our results may not be directly applicable to children currently being diagnosed with VUR. Fifth, rather than overall utility, which would include both efficacy and harm, we focused on assessing differences in efficacy. We did so because the trial lacked details to support in-depth analyses of harms related to antibiotic use [2]. Lastly, the assessment of BBD remains controversial. Our results are based on the methods used in RIVUR trial in which the Dysfunctional Voiding Symptoms Scale [18] was used to identify children ≥ 24 months of age with BBD.

In conclusion, our re-analysis of the RIVUR trial data suggests that the use of long-term antimicrobial prophylaxis appears to be most beneficial for children with BBD (and any grade of VUR) or those with grade IV VUR (regardless of BBD status).

## Figures and Tables

**Table 1:** Characteristics of the three subgroups that appear to benefit the most from long-term antimicrobial prophylaxis.

Subgroup Composition	Subgroup Size	Absolute Risk Difference (Placebo – Treatment)	Observed Number Needed to Treat
BBD & grade IV VUR	4	0.5–0 = 0.5	2^c^
BBD	71	0.51–0.18 = 0.33	4^b^
BBD or grade IV VUR^c^	117	0.47–0.19 = 0.27	4^d^

a Adjusted NNT of 2

b Adjusted NNT of 6

c Children with either BBD (and any grade VUR) or those with grade IV VUR (regardless of BBD status)

d Adjusted NNT of 7

## Key Points

### Question:

In children with vesicoureteral reflux, what combination of clinical and laboratory findings best predict children who benefit most from long-term antimicrobial prophylaxis?

### Findings:

Children with bowel-bladder dysfunction (irrespective of the grade of vesicoureteral reflux) or those with grade IV vesicoureteral reflux (irrespective of the presence of bowel-bladder dysfunction) appear to benefit the most from long-term antimicrobial prophylaxis.

### Meaning:

This study provides an evidence-based method of selecting children who benefit the most from long-term antimicrobial prophylaxis.
